# Supplementary material for: WordCommentsAnalyzer: A windows software tool for qualitative research
Source: F1000Res. 2018 Sep 4;7:536. Originally published 2018 May 3. [Version 2] doi: 10.12688/f1000research.14819.2 (PMC6137411; doi:10.12688/f1000research.14819.2)
Supplement: Supplementary file 3 [file f1000research-7-17614-s0002.tgz › b2a79496-4ada-47c4-9682-a99ce867836d.zip!/Eight Iranian Health Journals/Archives of Rehabilitation_49.docx]

© Archives of Rehabilitation. This work is licensed under CC BY-NC 4.0 (https://creativecommons.org/licenses/by-nc/4.0/).

Volume & Issue: دوره 8 - شماره 2

Title: بررسی تکرارپذیری معاینات بالینی و شاخص‌های رادیوگرافی در ارزیابی ناپایداری قطعه‌ای فقرات کمری

http://rehabilitationj.uswr.ac.ir/article-1-155-fa.html

هدف: تعیین تکرارپذیری اندازه‌گیریهای یک آزمونگر برای معاینات بالینی و شاخص‌های رادیوگرافی که جهت تشخیص بیماران دچار ناپایداری قطعه‌ای فقرات کمری استفاده می‌شوند هدف این مطالعه می‌باشد. روش بررسی: در این مطالعه توصیفی و متدلوژیک، 15 بیمار مبتلا به کمردرد مزمن غیر اختصاصی در محدوده سنی 43-22 سال با انتخاب غیر احتمالی و ساده شرکت داشتند. معاینات بالینی شامل سنجش شدت درد، دامنه حرکتی فلکسیون و اکستانسیون به روش شوبر، الگوهای حرکتی نابجا، آزمون تحمل فلکسوری و اکستانسوری به روش ایتو (Ito) و فلکسور جانبی با آزمون Sid Support، آزمون‌های بلند کردن مستقیم پا (SLR) دوطرفه و Sit-Up، آزمون Prone Instability و شاخص Oswestry بودند. متغیر‌های رادیوگرافی شامل متغیرهای کمیت حرکت (جابجایی و چرخش مهره‌ها در سطح ساجیتال) به دو روش دوپوئیس و پنجابی و متغیرهای کیفیت حرکت (فاکتور ناپایداری، مرکز چرخش و طول سنتروئید) بودند. از روش Putto برای اخذ رادیوگرافی فلکسیون – اکستانسیون استفاده شد. پنج کلیشه در وضعیت‌های نوترال، فلکسیون و اکستانسیون کامل و دامنه میانی فلکسیون و اکستانسیون از بیماران اخذ شد. برای تکرارپذیری متغیرهای کمی از ضریب همبستگی آی.سی.سی و در متغیرهای اسمی از آماره کاپا استفاده شد. یافته‌ها: نتایج حاکی از تکرارپذیری بالا در شدت درد (0/89=ICC)، دامنه حرکتی فلکسیون (0/93=ICC) و اکستانسیون (0/74=ICC)، آزمون پشتیبانی سید (ICC=0/85-0/86)، تحمل اکستانسوری (0/86=ICC) و فلکسوری (0/82=ICC) و شاخص اُسوِستری (0/97=ICC) است. ارزش کاپا برای الگوهای حرکتی نابجا (0/66=K)، اس.ال.آر دوطرفه و سیت‌آپ (K=0/65) و Prone Instability آزمون (K=0/66) نشاندهنده میزان توافق خوب می‌باشد. مختصات مرکز چرخش روی محور ایکس (ICC=0/99) و وای (ICC=0/96)، چرخش به روش دوپوئیس (0/83=ICC) و پنجابی (0/88=ICC) دارای تکرارپذیری بسیار بالا و طول سنتروئید با 3 کلیشه (0/60=ICC) و 5 کلیشه (0/64=ICC)، فاکتورناپایداری با 3 کلیشه (0/61=ICC) و 5 کلیشه (0/72=ICC)، جابجایی به روش دوپوئیس (0/69=ICC) و پنجابی (0/76=ICC) از تکرارپذیری متوسط به بالا برخوردارند. نتیجه‌گیری: آزمون‌های بالینی شامل شدت درد، دامنه حرکتی فلکسیون و اکستانسیون، آزمون Sid support، تحمل اکستانسوری و فلکسوری و شاخص Oswestry از تکرارپذیری بالا تا بسیار بالا برخودارند. الگوهای حرکتی نابجا، SLR دوطرفه و Sit-Up و آزمون Prone Instability از میزان توافق خوب برخوردارند. شاخص‌های رادیوگرافی دارای تکرارپذیری متوسط تا بسیار بالا هستند.

Title: بررسی پایایی مقیاس تعادلی برگ در ارزیابی تعادل کودکان فلج مغزی اسپاستیک

http://rehabilitationj.uswr.ac.ir/article-1-159-fa.html

هدف: ارزیابی تعادل در بیماران نورولوژیک مقدم بر برنامه‌ریزی درمانی می‌باشد. با مرور بر اطلاعات تست‌های تعادلی مشاهده می‌شود که اکثر آنها برای بیماران بزرگسال هنجاریابی شده‌اند و کمتر قابل استفاده در جمعیت کودکان می‌باشند. بنابراین دسترسی به مقیاسی پایا به منظور ارزیابی تعادل عملکردی در کودکان فلج مغزی ضروری می‌باشد. هدف این تحقیق بررسی پایایی اینترریتر و اینترا ریتر مقیاس تعادلی برگ در ارزیابی تعادل کودکان فلج مغزی اسپاستیک می‌باشد. روش بررسی: در این مطالعه توصیفی که از نوع اعتبارسنجی می‌باشد، 20 کودک مبتلا به فلج مغزی اسپاستیک با دامنه سنی 6 تا 12 سال و با میانگین سنی و انحراف معیار (X=7/35, SD=1/84)، با انتخاب غیر تصادفی در مطالعه شرکت نمودند. پس‌از کسب رضایت از والدین و آشنایی کودک با آزمون، ارزیابی اینترریتر در یک جلسه توسط آزمونگران بصورت تصادفی انجام و ارزیابی اینترا ریتر، یک هفته بعد انجام شد. از آزمون ضریب همبستگی درون طبقه‌ای (ICC) جهت همبستگی بین نمرات دو آزمونگر استفاده شد. یافته‌ها: با توجه به ضرایب همبستگی بدست آمده، توافق دو آزمونگر (P<0/001, ICC=0/985) و توافق در یک آزمونگر (P<0/001, ICC=0/094) عالی بود.  نتیجه گیری: مقیاس تعادلی برگ دارای پایایی اینترریتر و اینترا ریتر بالا (High) برای اندازه گیری تعادل در کودکان مبتلا به فلج مغزی اسپاستیک می‌باشد. بنابراین به عنوان یک معیار کلینیکی پایا برای ارزیابی تعادل این کودکان پیشنهاد می‌شود.

Title: مقایسه اثر تیپینگ مچ پا بر روی ثبات پاسچرال زنان مبتلا به بی ثباتی عملکردی مچ پا با زنان سالم

http://rehabilitationj.uswr.ac.ir/article-1-160-fa.html

هدف: بررسی مقایسه‌ای تأثیر تیپینگ مچ پا بر روی ثبات پاسچرال در بیماران مبتلا به بی‌ثباتی عملکردی مچ پا با افراد سالم هدف این تحقیق می‌باشد. روش بررسی: در مرحله اول یک مطالعه مقدماتی شامل تحقیق متدولوژیک جهت تعیین سطح تکرارپذیری اندازه‌های بدست آمده، در دو گروه 8 نفره از بیماران و افراد سالم به انجام رسید. نتایج حاکی از عالی بودن سطح تکرارپذیری نسبی و مطلق شاخص‌های ثباتی در شرایط مختلف آزمون بود. در مرحله دوم طی یک مطالعه شبه تجربی از نوع مورد - شاهدی در دو گروه 15 نفره از بیماران و افراد سالم که غیر تصادفی و ساده و بصورت هدفمند انتخاب شده بودند، شاخص‌های ثباتی کلی، قدامی – خلفی و طرفی با استفاده از سیستم تعادلی بایودکس، در دو شرایط با و بدون تیپینگ مچ پا و ایستادن روی یک پا و دو پا، مورد بررسی قرار گرفت. داده‌های تحقیق با استفاده از روشهای آماری ضریب همبستگی، آنووا و آنالیز واریانس تجزیه و تحلیل شد. یافته‌ها: کاربرد تیپینگ در مقایسه با شرایط قبل از آن، در دو گروه سالم و بیمار، نشانگر کاهش معنی‌دار میزان شاخص‌های ثباتی در کلیه آزمون‌ها بود (P<0/001). نتیجه‌گیری: مشکلات کنترل پاسچر را می‌توان به عنوان جنبه‌های مهمی‌در ارزیابی و درمان بیماران مبتلا به بی ثباتی عملکردی مچ پا در نظر گرفت که نیاز به ارزیابی و درمان دارد. به نظر می‌رسد تیپینگ مچ پا از طریق افزایش درون داده‌های حس عمقی باعث بهبود کنترل پاسچرال پویا در زنان مبتلا به بی ثباتی عملکردی مچ پا می‌شود.

Title: یادگیری صریح یک مهارت ادراکی – حرکتی به دنبال سکته مغزی با استفاده از دست مبتلا

http://rehabilitationj.uswr.ac.ir/article-1-161-fa.html

هدف: بررسی یادگیری صریح در بیماران مبتلا به سکته مغزی یک طرفه و مقایسه آن با افراد سالم و تعیین نقش عوامل مؤثر بر یادگیری این بیماران هدف این مطالعه می‌باشد. روش بررسی: در این تحقیق تحلیلی مورد - شاهدی، از یک نرم افزار کامپیوتری جهت بررسی یادگیری حرکتی استفاده شده است که طی آن 4 مربع با رنگهای مختلف بر صفحه نمایشگر ظاهر می‌شد و داوطلب به محض مشاهده هر مربع باید کلید تعریف شده مرتبط با آن را فشار می‌داد. 15 بیمار مبتلا به سکته مغزی با انتخاب هدفمند و غیر تصادفی و 15 فرد سالم همسان که با گروه مورد جور شده بودند، در این تحقیق شرکت داشتند. در روز اول 8 بلوک حرکتی تمرین می‌شد. 24 ساعت بعد یک آزمون یادآوری از داوطلبین بعمل می‌آمد که شامل دو بلوک حرکتی با الگوی منظم بود. همه داوطلبین راست دست بوده و بیماران نیز دارای ابتلای سمت راست بوده و با همان سمت مبتلا تمرین را انجام می‌دادند و زمان سکانسها و بلوکهای حرکتی به عنوان متغیر تحقیق اندازه گرفته می‌شد. یافته‌ها: انجام بلوکهای تحقیق در بیماران با اختلاف معنی‌داری کندتر از افراد سالم بود (P<0/05). بین زنان و مردان چه در بیماران و چه در افراد سالم اختلاف معنی‌داری وجود نداشت. همچنین متغیرهای سن، جنس و سطح تحصیلات تأثیر قابل‌توجهی بر نتایج تحقیق نداشت (P>0/05). زمان انجام بلوکهای حرکتی در دفعات آخر نسبت به دفعات ابتدایی کاهش معنی‌داری در هر دو گروه داشت (P<0/05). نتیجه‌گیری: اگرچه بیماران مبتلا به سکته مغزی یک طرفه، درمجموع حرکت را کندتر از افراد سالم همسان انجام می‌دادند، اما یادگیری حرکتی که بصورت کاهش پایدار در زمان بلوکها تعریف شده بود، در هر دو گروه سالم و بیمار وجود داشت. به عبارت دیگر بیماران مبتلا به سکته مغزی در طی انجام حرکت با دست مبتلا قابلیت یادگیری حرکتی را دارا هستند.

Title: تهیه و بررسی روایی و اعتبار آزمون هجی‌کردن در دانش‌آموزان کلاس اول ابتدایی در تهران

http://rehabilitationj.uswr.ac.ir/article-1-163-fa.html

هدف: این مطالعه با هدف تهیه آزمون هجی کردن و تعیین روایی و اعتبار آن در پایه اول ابتدایی به منظور بررسی تأثیر طول کلمه بر خطاهای هجی کردن انجام شده است. روش بررسی: این مطالعه به روش توصیفی و از نوع ابزار سازی و اعتبارسنجی می‌باشد. جمعیت مورد مطالعه دانش‌آموزان کلاس اول ابتدایی (یک کلاس از مدرسه دخترانه و یک کلاس از مدرسه پسرانه) از منطقه 8 آموزش و پرورش شهر تهران می‌باشند که به صورت آسان و غیر تصادفی انتخاب شدند. ابتدا کلمات کتاب «بنویسیم» پایه اول ابتدایی بر اساس طول کلمه (ساختار هجایی) مورد بررسی قرارگرفت. سپس از میان 494 کلمه موجود در کتاب فارسی، 241 کلمه که حاوی تمام ساختارهای هجایی بودند، برای انتخاب به صاحب نظران داده شد. بر اساس نظر آنان از میان 241 کلمه، 40 کلمه انتخاب گردید. این کلمات توسط دو آزمونگر در طی دو مرحله به تفکیک به دانش‌آموزان پایه اول ابتدایی به صورت دیکته گفته شد و خطاهای دیکته تعیین و با استفاده از آزمونهای آماری ضریب همبستگی پیرسون و تی‌زوجی مورد تجزیه و تحلیل آماری قرار گرفت.  یافته‌ها: نتایج نشان می‌دهد که ضریب همبستگی بین اجراهای متوالی بالا است. همچنین مقایسه میانگین تعداد خطاها در نوبت‌های مختلف اجرا نشان می‌دهد که در پسران اختلاف میانگین از نظر آماری معنی‌دار نیست (مقدار p به‌ترتیب در دو مرحله اجرا توسط آزمونگر اول و دوم عبارت است از: P=0/33, P=0/56)، اما در دختران بین میانگین تعدادخطاهای مرحله اول و دوم از نظر آماری اختلاف وجود دارد (مقدار p به ترتیب در دو مرحله اجرا توسط آزمونگر اول و دوم عبارت است از: P=0/002, P=1). نتیجه‌گیری: این یافته‌ها نشان می‌دهد آزمون مورد نظر از روایی و اعتبار بالایی برخوردار است و می‌توان از آن در پژوهشهای مورد نظر و بررسی ارتباط ساختار هجایی کلمه و خطاهای هجی کردن استفاده نمود.

Title: بررسی و مقایسه کنش‌پریشی در سالمندان مبتلا به دمانس نوع آلزایمر و سالمندان سالم

http://rehabilitationj.uswr.ac.ir/article-1-164-fa.html

هدف: بسیاری از بیماران مبتلا به آلزایمر در مراحل اولیه بیماری، علاوه بر نقایص حافظه و زبان پریشی، کنش پریش هستند که می‌تواند باعث بروز اختلالاتی در زندگی روزمره آنان گردد. هدف از تحقیق حاضر بررسی وجود هرگونه کنش‌پریشی و نوع آن در سالمندان مبتلا به دمانس نوع آلزایمر در مراحل اولیه و مقایسه آن با گروه سالمندان سالم (کنترل) می‌باشد. روش بررسی: این تحقیق از نوع تحلیلی مورد-شاهدی می‌باشد. تعداد 14 بیمار سالمند مبتلا به دمانس از نوع آلزایمر و 20 نفر سالمند سالم که از نظر سن و تحصیلات با آنها همتا شده بودند مورد مطالعه قرار گرفتند. بیماران از بین مراجعان به انجمن آلزایمر و با تشخیص روانپزشک از بین افراد با دمانس خفیف تا متوسط (MMSE=15-27) بصورت هدفمند انتخاب شده و با سالمندان سالم (30-25=MMSE) مقایسه گردیدند. روی افراد هر دو گروه آزمون کنش‌پریشی اجرا گردید. این آزمون شامل چهار زیرآزمون حرکات مفهومی، حرکت‌های مفهومی اشاره با دست، حرکت‌های دهانی و تنفسی و حرکت‌های تقلیدی کار با اشیا می‌باشد. داده‌ها با استفاده از آزمون کولموگروف – اسمیرنوف، من – ویتنی، آزمون تی و ضریب همبستگی پیرسون مورد تجزیه و تحلیل قرار گرفت. یافته‌ها: نتایج نشان داد که نمرات آزمون کنش‌پریشی در دو گروه بیمار و کنترل تفاوت معنی‌داری دارد (P<0/001). به طوری که بیماران آلزایمر، مبتلا به کنش‌پریشی و افراد سالمند سالم، غیر مبتلا به کنش‌پریشی تشخیص داده شدند. علاوه بر این مقایسه نمرات افراد بیمار در زیر آزمون‌های مختلف آشکار کرد که نمرات آنان در زیر آزمون حرکات مفهومی از همه زیرآزمون‌های دیگر کمتر (کنش‌پریشی بیشتر) و تفاوت آن معنادار می‌باشد (P<0/001). همچنین نمرات بیماران در زیرآزمون حرکت‌های دهانی و تنفسی از همه زیرآزمون‌ها بیشتر (کنش‌پریشی کمتر) و تفاوت آن معنادار است (P<0/001). نتیجه‌گیری: کنش‌پریشی به عنوان یکی از علائم عصب روانشناختی در مراحل اولیه بیماری آلزایمر قابل بررسی است و می‌تواند به صورت تکمیلی برای تأیید تشخیص افتراقی به کار رود. بررسی زیر گروه‌های کنش‌پریشی نیز می‌تواند در تعیین نوع کنش‌پریشی، فهم اختلالات مشاهده شده و راه‌های احتمالی توان بخشی آنان کمک کند.

Title: تأثیر مداخلات کاردرمانی بر مهارت‌های بینایی-حرکتی کودکان دارای اختلال ویژه یادگیری در مقطع ابتدایی

http://rehabilitationj.uswr.ac.ir/article-1-165-fa.html

هدف: بررسی و تشخیص اختلال بینایی-حرکتی در کودکان با اختلال ویژه یادگیری و کاربرد مداخلات کاردرمانی در جهت ارتقا و بهبود این مهارت، می‌تواند در بهبود وضعیت خواندن و نوشتن این کودکان مؤثر واقع شود. هدف از مطالعه حاضر، بررسی تأثیر مداخلات کاردرمانی بر مهارتهای بینایی- حرکتی کودکان دارای اختلال ویژه یادگیری در مقطع ابتدایی می‌باشد. روش بررسی: مطالعه حاضر به روش تجربی و مداخله‌ای می‌باشد. برای انجام این پژوهش، 23 کودک با اختلال ویژه یادگیری (پایه دوم، سوم و چهارم) از مراجعین به مرکز مشکلات ویژه یادگیری شهر تهران، انتخاب شدند. این کودکان از نظر بینایی و شنوایی سالم و بهره هوشی آنها طبیعی بود. نمونه‌ها بطور تصادفی به دو گروه مداخله (11 نفر) و مقایسه یا شاهد (12 نفر) تقسیم شدند. مهارتهای بینایی- حرکتی کلیه نمونه‌ها ابتدا توسط آزمون مهارتهای بینایی- حرکتی (TVMS-R) مورد ارزیابی قرار گرفت. سپس مداخلات کاردرمانی در گروه مداخله صورت گرفت و مجدداً توسط آزمون TVMS-R ارزیابی هر دو گروه انجام و با استفاده از آزمون تی زوجی و تی مستقل مورد تجزیه و تحلیل آماری قرار گرفت. یافته‌ها: در بررسی نمره کل آزمون، اختلاف معناداری در گروه مقایسه، قبل و بعد از مداخله داده نشد (P=0/4)، اما در گروه مداخله اختلاف معناداری دیده شد (P<0/001). در بررسی مجموع نمرات طبقه 2، 5 و 7 نیز که علاوه بر مهارتهای یکپارچگی بینایی- حرکتی و تثبیت چشمی بیشتر نیازمند مهارتهای بینایی- فضایی هستند، تأثیر معنادار مداخلات کاردرمانی دیده شد (P<0/001) و بررسی مجموع نمرات طبقات 1، 3، 4، 6 و 8 نشان داد که مداخلات کاردرمانی تأثیر معناداری بر مجموع این طبقات و در نتیجه مهارتهای تجزیه و تحلیل بینایی داشته است (P<0/005). نتیجه گیری: مداخلات کاردرمانی بر مهارتهای بینایی- حرکتی و اجزای آن (یکپارچگی بینایی- حرکتی، تجزیه و تحلیل بینایی و بینایی- فضایی و تثبیت چشمی) در کودکان دچار اختلال ویژه یادگیری مراجعه کننده به مرکز مورد نظر، بطور معناداری مؤثر بوده است.

Title: تعیین ضریب پایایی دستورالعمل کنترل حرکتی دهان گفتار رابینز-کلی در جامعه کودکان فارسی زبان اصفهانی و ارائه برخی از نمرات هنجار آنها

http://rehabilitationj.uswr.ac.ir/article-1-166-fa.html

هدف: بررسی حاضر با هدف ارائه یک ارزیابی جامع و کامل از ساختمان و عملکرد حرکتی مکانیسم گفتار در کودکان پیش‌دبستانی شکل گرفت. روش بررسی: این مطالعه در بخش اول بصورت توصیفی و اعتبارسنجی و در بخش دوم بصورت مقطعی مقایسه‌ای انجام شد. پس از ترجمه و اعتباریابی پروتکل کنترل حرکتی دهان-گفتار رابینز و کلی طی یک مطالعه مقدماتی، این دستورالعمل به منظور ارزیابی عملکرد راه صوتی برای 300 کودک 3 تا 6 ساله فارسی زبان اصفهانی که به شیوه تصادفی و خوشه‌ای از بین مهدکودکها و آمادگیها انتخاب و به سه گروه تقسیم شده بودند، اجرا شد. از معیار آلفای کرونباخ برای تعیین ضریب پایایی و برای گزارش هنجارها از ارائه میانگین و انحراف معیار استفاده شده است. برخی از تفاوتهای مشاهده شده میان گروههای سنی مختلف نیز با آزمونهای تی و آنووا مورد تحلیل قرار گرفت.  یافته‌ها: نمرات کودکان 5 تا 6 ساله در اغلب متغیرهای عملکردی به طور معناداری بیشتر از سایر گروههای سنی بود (P<0/001). ضریب پایایی فرم پروتکل نیز به تفکیک گروههای سنی و دو متغیر ترکیبی نمره کل ساختمان از 0/19 تا 0/59 و ‌نمره‌کل عملکرد اندامهای گفتاری از 0/75 تا 0/80 گزارش شد. نتیجه‌گیری: عملکردهای گفتاری کودکان با افزایش سن افزایش یافته که نشان از بلوغ عصبی و مهارتی کودکان بزرگتر در اجرای عملکردهای دهانی و گفتاری دارد. این پروتکل در مقایسه با سایر ارزیابی‌های موجود می‌تواند ارزیابی صحیح تری از مکانیسم دهان- حنجره در دیزارتری دوران کودکی ارائه داده و برای آپراکسی رشدی گفتار نیز معرف بهتری نسبت به سایر تستهای موجود می‌باشد. با توجه به بالا بودن ضرایب پایایی ‌نمره‌کل عملکرد اندامهای گفتاری در گروههای مختلف سنی، می‌توان از این بخش از پروتکل در تحقیقات و امور بالینی استفاده مطلوب نمود.

Title: سنجش میزان اثر بخشی توانبخشی روانی بر کودکان دارای معلولیت جسمی و ذهنی

http://rehabilitationj.uswr.ac.ir/article-1-167-fa.html

هدف: با توجه به اینکه بومی کردن مدل‌های توانبخشی از اهمیت خاصی برخوردار است؛ در این مطالعه ضمن معرفی توانبخشی روانی ژاپنی، میزان اثر بخشی آن بر کودکان دارای معلولیت جسمی و ذهنی سنجیده می‌شود.  روش بررسی: در این پژوهش شبه تجربی از نوع قبل و بعد، با انتخاب 120 نفر کودک 5 تا 10 ساله دارای معلولیت‌های فلج‌مغزی (CP) و کم‌توان ذهنی (MR) بصورت نمونه‌های در دسترس از کلینیکهای توانبخشی تهران، با انجام تکنیک‌های توانبخشی روانی به روش ژاپنی (دوسا) به مدت سه ماه شامل 24 جلسه یکساعته درمانی برای هر کودک و جمعاً 2880 جلسه درمانی، وضعیت قبل و بعد از مداخله کودکان مورد مقایسه قرار گرفت. کودکان مبتلا به فلج مغزی جهت بررسی تأثیر انجام تکنیکهای دوسا بر وضعیت ستون فقرات و متغیرهای قابل سنجش قوس گردن، پشت، کمر و وضعیت لگن و کودکان کم‌توان ذهنی جهت بررسی تأثیر انجام تکنیکهای دوسا بر وضعیت بدن و حرکت و چکونگی تعامل اجتماعی آنها، مورد مطالعه قرار گرفتند. ابزار پژوهش شامل کیت سنجش، پرسشنامه تعامل اجتماعی و ابزارتصویری ترسیمی بود. داده‌های حاصل با انجام آزمونهای آماری ویلکاکسون و آنووا مورد تجزیه تحلیل قرار گرفت. یافته‌ها: تکنیکهای درمانی دوسا بطور معناداری بر بهبود وضعیت ستون فقرات و قوسهای گردن، پشت و کمر کود کان مبتلا به فلج مغزی مؤثر بوده است (P<0/05). همچنین در مورد کودکان کم‌توان ذهنی، وضعیت بدن و حرکت آنان بعد از مداخله با توجه به نتیجه آزمون آنووا نسبت به قبل از مداخله اختلاف معناداری نشان نداد (P=0/237). تعامل اجتماعی آنان بخصوص تعامل با خانواده بعد از تمرینات پیشرفت چشمگیری داشت (P<0/001). نتیجه‌گیری: بطور کلی با توجه به شواهد آماری، تصویری و بالینی این تحقیق می‌توان نتیجه گرفت که تمرینات برنامه توانبخشی روانی دوسا بر بهبود وضعیت بدنی و روانی کودکان مبتلا به فلج مغزی و کم‌توان ذهنی مؤثربوده است و می‌توان از آن بعنوان یک روش تکمیلی در درمان استفاده کرد.

Title: رابطه تحلیل فضایی با دست‌نویسی در دانش‌آموزان کم‌توان ذهنی شهرری (1385)

http://rehabilitationj.uswr.ac.ir/article-1-168-fa.html

هدف: یکی از مشکلات رایج در مدارس کودکان کم‌توان ذهنی اختلالات دست‌نویسی است. هدف از این پژوهش بررسی ارتباط تحلیل فضایی و دست‌نویسی در دانش‌آموزان کم‌توان ذهنی است. روش بررسی: در این پژوهش که از نوع مطالعات توصیفی مقطعی و به روش همبستگی است، کلیه 126 دانش‌آموز کم‌توان‌ذهنی (53 دختر و 73 پسر) در دامنه سنی 9 تا 19 سال که در سال تحصیلی 85-1384 در پایه‌های اول تا پنجم ابتدایی در مدارس استثنایی شهرری مشغول به تحصیل بودند، به روش تمام شمار شرکت نمودند. برای همه دانش‌آموزان چک لیست دست‌نویسی محقق ساخته و نیز خرده‌آزمون طراحی با مکعب‌های وکسلر اجرا شد. داده‌ها با استفاده از آزمونهای آماری تی مستقل، یومن ویتنی، کروسکال والیس و همبستگی اسپیرمن مورد تجزیه و تحلیل قرار گرفتند. یافته‌ها: ضریب همبستگی ثانوی کندال نوع C نشان داد که بین دست‌نویسی و تحلیل فضایی رابطه مثبت معنی‌دار وجود دارد (r=0/305, P<0/0001). همچنین تحلیل فضایی با اندازه حروف نسبت به هم (P<0/007)، با اندازه کل نوشته (P<0/0001) و با روی خط نویسی (P<0/0002) رابطه مثبت معنی‌دار دارد. نتیجه‌گیری: وجود ارتباط بین تحلیل فضایی و دست‌نویسی مبین این امر است که به منظور بهبود خوانایی دست‌نویسی دانش‌آموزان کم‌توان ذهنی در مقوله‌های اندازه حروف نسبت به هم، اندازه کل نوشته و روی خط نویسی، مداخلات درمانی در زمینه تحلیل فضایی الزامی است.

Title: نقش حافظه آشکار و حافظه ضمنی در افراد مبتلا به لکنت

http://rehabilitationj.uswr.ac.ir/article-1-169-fa.html

هدف: لکنت یکی از شایع ترین اختلالات گفتاری است. با این وجود علت آن به درستی مشخص نیست. آسیب عملکردهای شناختی همچون حافظه هیجانی و توجه یکی از عوامل مهم آن به شمار می‌رود. در این تحقیق بعنوان هدف اصلی، عملکرد حافظه آشکار و حافظه ضمنی بین افراد مبتلا به لکنت و افراد عادی مورد مقایسه قرار گرفته و همچنین بصورت جنبی میزان اضطراب و افسردگی در این دو گروه مقایسه شده است. روش بررسی: این مطالعه ازنوع تحلیلی مورد-شاهدی تحلیلی می‌باشد. تعداد 30 نفر افراد مبتلا به لکنت به عنوان آزمودنی با انتخاب هدفمند از نمونه‌های در دسترس و مراجعه کننده به کلینیک‌های گفتار درمانی تهران و 30 نفر افراد عادی به عنوان گروه شاهد از طریق همتاسازی با گروه مورد بر اساس سن، جنس، تحصیلات و دوزبانگی مورد مقایسه قرار گرفتند. برای بررسی عملکرد حافظه آشکار از آزمون یادآوری نشانه‌ای و برای بررسی عملکرد حافظه ضمنی از آزمون تکمیل کردن ریشه واژه و برای سنجش میزان اضطراب و افسردگی افراد از پرسشنامه سلامت عمومی (28 GHQ) استفاده شد. سپس متغیرهای تعداد واژه‌های با بار عاطفی مثبت و منفی در حافظه آشکار و حافظه ضمنی و نیز نمره میزان اضطراب و افسردگی با استفاده از آزمونهای آماری تی مستقل، تی زوجی، من ویتنی و ویلکاکسون مورد تجزیه و تحلیل قرار گرفت. یافته‌ها: نتایج نشان داد که افراد مبتلا به لکنت واژه‌های با بار عاطفی مثبت درحافظه آشکار را به طور معنی‌داری نسبت به افراد عادی کمتر بازشناسی می‌کنند. همچنین افراد مبتلا به لکنت در آزمون‌های حافظه آشکار وحافظه ضمنی، واژه‌های با بار عاطفی منفی را بیشتر از واژه‌های با بار عاطفی مثبت بازشناسی می‌کنند (P<0/05). علاوه بر این مشخص شد که میزان اضطراب افراد مبتلا به لکنت به طور معناداری بیشتر از افراد عادی بود (P<0/001)، همچنین میزان افسردگی افراد مبتلا به لکنت بیشتر از افراد عادی بود ولی تفاوت آنها معنادار نبود (P<0/05). نتیجه‌گیری: با توجه به نقش عملکردهای شناختی از جمله حافظه هیجانی در برنامه‌ریزی حرکتی گفتار و مشاهده تفاوت درعملکرد حافظه هیجانی مثبت و منفی بین افراد لکنتی و عادی در این مطالعه، به نظر می‌رسد که توجه به مقوله حافظه هیجانی در بازتوانی افراد مبتلا به لکنت می‌تواند مؤثر واقع شود.

Title: تأثیر تکنیک آرام‌سازی پیشرونده عضلانی بر فعالیت‌های روزمره زندگی در بیماران مبتلا به مولتیپل‌اسکلروزیس

http://rehabilitationj.uswr.ac.ir/article-1-170-fa.html

هدف: بیماری مولتیپل اسکلروزیس یکی از شایعترین بیماریهای سیستم عصبی مرکزی با ماهیت مزمن و غیر قابل پیش بینی و روندی رو به افزایش است. هدف از این مطالعه بررسی تأثیر تکنیک آرامسازی پیشرونده عضلانی بر فعالیتهای روزمره زندگی بیماران مبتلا به مولتیپل‌اسکلروزیس می‌باشد. روش بررسی: این پژوهش یک مطالعه مداخله‌ای و تجربی است که پس از انتخاب 66 نفر با نمونه‌گیری هدفمند و به روش در دسترس، نمونه‌ها به صورت تصادفی به دو گروه شاهد و آزمون تخصیص داده شدند. پس از یک جلسه آشنا‌سازی نمونه‌ها با اهداف و روش مداخله و 63 جلسه اجرای تکنیک توسط گروه آزمون در مدت دو ماه، درحالیکه برای گروه شاهد هیچگونه مداخله‌ای انجام نشده بود، دو گروه مورد مقایسه قرار گرفتند. ابزارهای جمع‌آوری اطلاعات شامل پرسشنامه اطلاعات دموگرافیک، پرسشنامه فعالیتهای روزمره زندگی وچک لیست‌های خود گزارش دهی بود. داده‌‌های خام با آزمونهای آماری تی زوجی، تی مستقل، آنالیز واریانس و تعیین همبستگی مورد تجزیه و تحلیل قرار گرفتند. یافته‌ها: بر اساس نتایج حاصل، تفاوت معنی‌داری بین میانگین امتیاز کل و امتیاز ابعاد فعالیتهای روزمره زندگی قبل و بعد از مداخله در گروه آزمون وجود داشت (P>0/05)، ولی در مورد گروه شاهد این تفاوت معنی‌دار نمی‌باشد. بین میانگین امتیاز کل و امتیازات ابعاد مختلف فعالیتهای روزمره زندگی، یک ماه و دو ماه بعد از مداخله اختلاف معنی‌داری بین دو گروه شاهد و آزمون دیده شد (P<0/05). همچنین تفاوت معنی‌داری بین میانگین امتیازات بعد اجتماعی فعالیتهای روزمره زندگی در سه بار اندازه‌گیری بین دو گروه بدست آمد (P<0/02). در حالیکه این آزمون بین میانگین امتیازات سه بعد دیگر (فیزیکی، حسی، شخصی) فعالیتهای روزمره زندگی و امتیاز کل تفاوت معنی‌داری را نشان نمی‌دهد (P&ge0/095). نتیجه‌گیری: با توجه به یافته‌های این پژوهش می‌توان نتیجه گرفت که اجرای تکنیک آرامسازی پیشرونده عضلانی در بیماران مبتلا به مولتیپل‌اسکلروزیس عملی است و اجرای این تکنیک باعث ارتقاء سطح فعالیتهای روزمره زندگی این بیماران می‌گردد.

Title: بررسی اجمالی اثرات مانیپولاسیون و موبیلیزاسیون ستون فقرات

http://rehabilitationj.uswr.ac.ir/article-1-171-fa.html

مانیپولاسیون و موبیلیزاسیون دو شکل متفاوت از درمانهای دستی محسوب شده که بطور شایع در درمان اختلالات عضلانی -اسکلتی مورد استفاده قرار می‌گیرند. تشخیص افتراقی این دو روش درمانی اغلب با استناد به پارامترهای بیومکانیکی خاصی نظیر حداکثر نیرو، مدت زمان و میزان جابجایی انجام می‌پذیرد. البته تابحال تحقیقات کمی برروی افتراق دو روش مزبور از نقطه نظر سازوکارهای نورولوژیک یا کارآیی بالینی صورت گرفته است. مانیپولاسیون به ویژه موجب تحریک گیرنده‌های موجود در عضلات بین مهره‌ای عمقی گشته، در حالیکه روشهای موبیلیزاسیون اکثراً برروی عضلات محوری سطحی تأثیر می‌گذارند.

Title: برنامه‌های مداخله به هنگام و شیرخواران پرخطر

http://rehabilitationj.uswr.ac.ir/article-1-172-fa.html

سرویس‌های مداخله به هنگام خدمات تشخیصی-درمانی درکودکان در معرض تکامل غیر طبیعی در سالهای اول زندگی هستند که در مواجهه با ناتوانی‌های مشخص یا عوامل خطرساز طبی و روانشناختی همانند نارس بودن یا بی توجهی والدین می‌باشند. در این بحث به انواع ساختار بین بخشی و برنامه‌های پیگیری شیرخواران پر خطر اشاره ‌شده و نظرات رایج در خصوص اثربخشی این روندها در پیشرفت تکامل کودک عنوان می‌گردد. نقش متخصص کودکان در تیم پیگیری به عنوان مراقب، ارجاع دهنده، هماهنگ‌کننده تیم مداخله و در پاسخگویی به کودک- خانواده مطرح است.
